# HTLV-I antisense transcripts initiating in the 3'LTR are alternatively spliced and polyadenylated

**DOI:** 10.1186/1742-4690-3-15

**Published:** 2006-03-02

**Authors:** Marie-Hélène Cavanagh, Sébastien Landry, Brigitte Audet, Charlotte Arpin-André, Patrick Hivin, Marie-Ève Paré, Julien Thête, Éric Wattel, Susan J Marriott, Jean-Michel Mesnard, Benoit Barbeau

**Affiliations:** 1 Centre de Recherche en Infectiologie, Centre Hospitalier Universitaire de Québec, Pavillon CHUL, and Département de Biologie médicale, Faculté de Médecine, Université Laval, Ste-Foy (Québec), G1V 4G2, Canada; 2 Laboratoires Infections Rétrovirales et Signalisation Cellulaire, CNRS/UM I UMR 5121/IFR 122, Institut de Biologie, 34960 Montpellier Cedex 2, France; 3 Oncovirologie et Biothérapies, UMR5537 CNRS-Université Claude Bernard, Centre Léon Berard and Service d'Hématologie, Pavillon E, Hôpital Edouard Herriot, Place d'Arsonval, Lyon, France; 4 Department of Molecular Virology and Microbiology, Baylor College of Medicine, Houston, Texas 77030, USA; 5 Université.du Québec à Montréal, Département des sciences biologiques, C.P. 8888, Succursale C.V., Montréal, Québec, H3C 3P8, Canada

## Abstract

**Background:**

Antisense transcription in retroviruses has been suggested for both HIV-1 and HTLV-I, although the existence and coding potential of these transcripts remain controversial. Thorough characterization is required to demonstrate the existence of these transcripts and gain insight into their role in retrovirus biology.

**Results:**

This report provides the first complete characterization of an antisense retroviral transcript that encodes the previously described HTLV-I HBZ protein. In this study, we show that HBZ-encoding transcripts initiate in the 3' long terminal repeat (LTR) at several positions and consist of two alternatively spliced variants (SP1 and SP2). Expression of the most abundant HBZ spliced variant (SP1) could be detected in different HTLV-I-infected cell lines and importantly in cellular clones isolated from HTLV-I-infected patients. Polyadenylation of HBZ RNA occurred at a distance of 1450 nucleotides downstream of the HBZ stop codon in close proximity of a typical polyA signal. We have also determined that translation mostly initiates from the first exon located in the 3' LTR and that the HBZ isoform produced from the SP1 spliced variant demonstrated inhibition of Tax and c-Jun-dependent transcriptional activation.

**Conclusion:**

These results conclusively demonstrate the existence of antisense transcription in retroviruses, which likely plays a role in HTLV-I-associated pathogenesis through HBZ protein synthesis.

## Background

Natural antisense transcription has been described in several eukaryotic organisms and has been ascribed several functions [1-3]. Retroviruses have long been thought to lack antisense transcription and to rely on a single sense transcript for viral gene expression. Unspliced and spliced sense transcripts are thought to produce all viral proteins required for replication and survival in the infected host. Although a few studies have suggested that retroviruses might produce antisense transcripts with coding potential [4-10], the existence of such atypical RNAs has not been conclusively demonstrated. Recent identification of the HBZ (HTLV-I bZIP) protein, surprisingly encoded on the antisense strand of human T-cell leukemia virus type I (HTLV-I), revived the likely existence of antisense transcription among retroviruses [11].

HTLV-I is the etiological agent of adult T cell leukemia/lymphoma (ATLL) and HTLV-I-associated myelopathy (also termed tropical spastic paraparesis) (HAM/TSP) [12-17]. In the sense strand, the HTLV-I genome encodes typical retroviral proteins as well as other more HTLV-I-specific proteins, such as Tax. The viral Tax protein has been suggested to play an important role in the diseases occurring in HTLV-I-infected patients. Tax is an important transactivator and acts upon the HTLV-I gene expression by promoting protein complexes involving CREB and the CREB binding Protein (CBP) on the TRE1 regions present in the HTLV-I long terminal repeat (LTR) promoter region.

**Figure 1:**
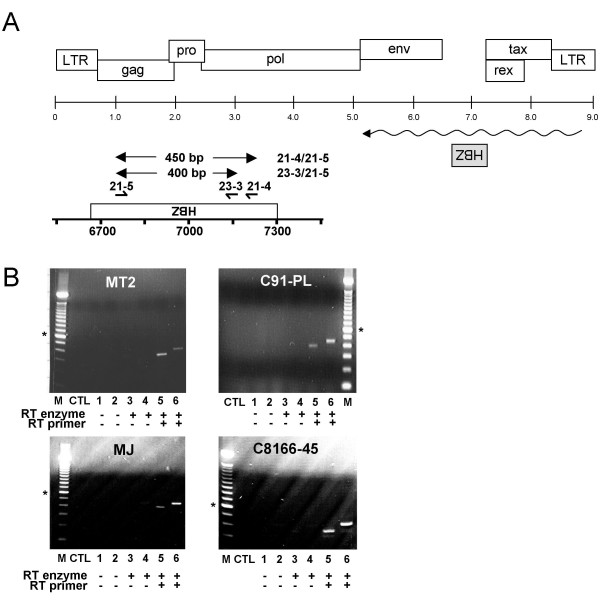
Detection of the HTLV-I antisense transcript in HTLV-I-infected cell lines. (***A***) Positioning of the HBZ antisense ORF in the HTLV-I proviral DNA. Primers used for RT-PCR experiments and the expected size of the amplified signal are indicated above the enlarged HBZ ORF. (***B***) RT-PCR analyses were performed on RNA samples from HTLV-I-infected cell lines using the 21-5 primer for RT and primer combinations presented in *A *for PCR analysis. Samples were tested for DNA contamination in RNA samples (lanes 1–2; no RT and no RT primer) and autopriming (lanes 3–4; in the presence of RT with no added RT primer). CTL represents PCR analysis with no added cDNA or RNA. M = 100 bp marker (the asterisk indicates the 600 bp band). Lanes 5 and 6 show the results of PCR using primers 23-3/21-5 and 21-4/21-5 to generate products of 400 bp and 450 bp, respectively.

Upon its discovery, the HBZ-coding region has been shown to be located between Tax exon 3 and Env exon 2 in the antisense strand (see Fig. 1A) [11]. The HBZ protein possesses peculiar functions, which suggest that this viral protein could have a potential impact on HTLV-I-associated pathogenesis. Specifically, the HBZ protein can inhibit Tax activation of both AP-1 function and HTLV-I LTR-mediated gene expression through various protein-protein interactions [11,18-20]. A recent study by Arnold *et al*. [21] have demonstrated that, although HBZ was dispensable for viral replication in cell culture, persistence of HTLV-I in inoculated rabbits was enhanced by HBZ. Although several reports have characterized functions of the HBZ protein, the structure of its transcript and the mechanisms behind HBZ gene regulation remain poorly-defined. Complete characterization of the HBZ transcript is critical to conclusively demonstrate that antisense transcription is a mechanism of retroviral gene expression.

In this report, we have focussed on the characterization of the HBZ-encoding antisense transcript produced from the HTLV-I genome. Our results show that HBZ-encoding transcripts initiate in the 3' LTR, are polyadenylated and are alternatively spliced. Furthermore, the HBZ isoform produced from the most abundant spliced form possesses similar functional properties to the one previously attributed to the former HBZ isoform. These results will strongly impact the field of retrovirology, being the first clear demonstration of the existence of antisense transcription in retroviruses.

## Results and discussion

### Detection of the antisense transcript in transfected 293T cells and HTLV-I-infected cell lines

The identification of the HBZ gene has raised several important issues regarding the various mechanisms governing retroviral gene expression. Its atypical positioning in the HTLV-I genome (Fig. 1A) warranted further investigation and a more thorough characterization of the HBZ-encoding RNA was thus conducted.

**Figure 2:**
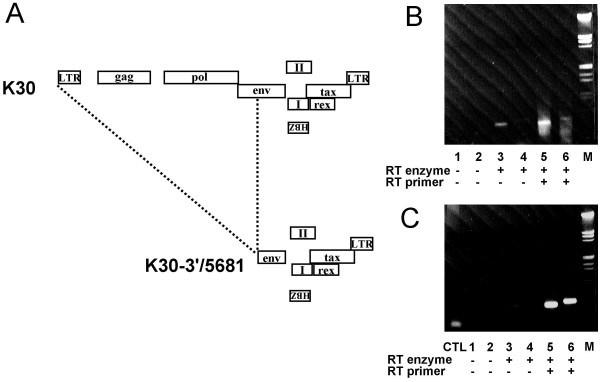
Detection of the HTLV-1 antisense transcript in HTLV-I-producing 293T cells. **(*A*) **K30 and K30-3'/5681 proviral DNA constructs are depicted. The deleted region for the latter construct is shown. ***(B-C) ***293T cells were transfected with 5 μg K30 **(*B*) **or K30-3'/5681 **(*C*)**. RT-PCR analyses was then conducted on RNA isotated from transfected 293T cells. RT-PCR conditions and controls were performed as in fig. 1. M = lambda DNA (EcoRI/HindIII) marker.

Our first objective was to specifically demonstrate that HTLV-I indeed produced antisense transcripts using RT-PCR. Negative controls were carefully selected to avoid previously reported autopriming artifacts that can occur during the reverse transcription step of RT-PCR analysis [7,22]. RT reactions were either performed without primer (control for autopriming) or with a primer complementary to the deduced HBZ ORF sequence (see Fig. 1A). Additional controls included RNA samples in which the RT step had been omitted prior to PCR amplification. Using these controls, RT-PCR analyses were first performed using two sets of PCR primers specific for the HBZ-coding sequence. As demonstrated in Fig. 1B lanes 5 and 6, antisense HBZ transcripts were observed in all HTLV-I-infected cell lines tested, while similar signals were not observed in the various controls. To confirm the above results, RT-PCR analyses were next conducted in 293T cells transfected with the HTLV-I K30 molecular DNA proviral clone (Fig. 2A–B). The expected signal (although weak) was observed in transfected 293T cells. As demonstrated in lane 3 (Fig. 2B), autopriming was however apparent in K30-transfected 293T cells, likely due to high levels of sense RNA that is reverse transcribed independently of the HBZ-specific primer. To eliminate this artefact, sense transcription from the K30 proviral DNA was knocked out by deletion of the 5' end of the proviral genome (Fig. 2A–C). The resulting K30-3'/5681 construct was then transfected in 293T cells. RT-PCR analyses showed a stronger antisense-derived signal and no autopriming signal was observed, suggesting that sense RNAs were the source of the contaminating autopriming signal.

These results clearly demonstrated the existence of an antisense transcript in HTLV-I, which included the HBZ sequence. The use of HTLV-I proviral DNA clones and of infected cell lines demonstrated that a wide range of HTLV-I clones is capable of producing this transcript. Furthermore, data from the transfected 293T cells with the 5'LTR-deleted proviral DNA construct also argued that sense transcription could impede antisense transcription, which might be expected.

### HBZ transcripts initiate in the 3' LTR at different position

**Figure 3:**
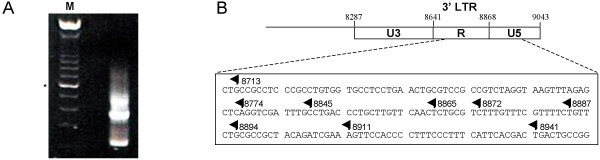
HTLV-I antisense transcription initiates in the 3' LTR. (***A***) 5'RACE analysis was conducted using RNA samples from 293T cells transfected with the K30-3'/5681 proviral DNA construct. The resulting amplified products were run on an agarose gel. M = 100 bp marker (the asterisk indicates the 600 bp band). (***B***) Position of the identified CAP sites for antisense transcripts are depicted in the 3' LTR. Nucleotide numbering corresponds to the sense strand.

We were then interested in determining the transcription initiation site of the HBZ transcript. RNA from transfected 293T cells was analysed using the 5'RLM-RACE kit. Final PCR amplification was conducted with reverse primers positioned near the 5' end of the HBZ-coding region and primers specific to the oligonucleotide ligated to the 5' end of RNAs. Cloning and sequencing of all amplified products generated by 5' RACE (Fig. 3A) identified several CAP sites positioned in the 3' LTR (exclusively in the R and U5 regions) and spanning a total of 228 nt (Fig. 3B). Frequently used transcription initiation sites were identified at positions 8713, 8865, 8887 and 8894.

These results hence demonstrated that the HBZ transcript initiated in the 3' LTR at multiple positions. This multiplicity of initiation sites might be a consequence of the absence of TATA boxes at close distance. Our results parallel the data presented on the localisation of the transcription initiation sites specific for HIV-1 antisense transcripts, which were near or in the 3' LTR region [6,7]. Similar to HIV-1, based on the positioning of the transcription initiation sites, it is expected that the promoter region for HTLV-I antisense transcription would be present in the 3'LTR region as initially suggested by Larocca *et al. *[4]. Further investigations are required to determine the mechanism of regulation of this promoter region and to evaluate the possible involvement of adjacent cellular DNA in these regulatory mechanisms.

### HBZ transcripts are alternatively spliced

**Figure 4:**
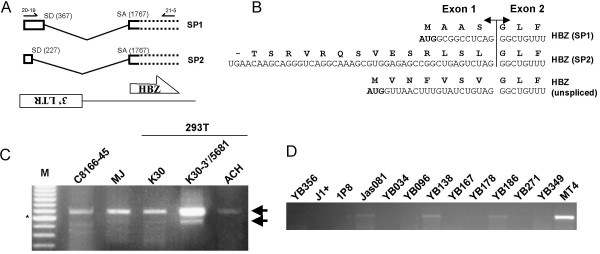
HBZ transcripts are alternatively spliced. (***A***) The position of splice junctions within the two HBZ SP1 and SP2 RNA are positioned relative to the 3'LTR and the HBZ ORF. Nucleotide numbering corresponds to the antisense strand. (***B***) Predicted amino acid sequences for all potential HBZ isoforms are shown above each cDNA sequence. Sequences from exons 1 and 2 are separated and identified accordingly. The AUG initiation codon in unspliced and SP1 HBZ RNAs are highlighted in bold. (***C***) RNA isolated from HTLV-I-infected cell lines and 293T cells transfected with 5 μg K30, K30-3'/5681 or ACH was analyzed by RT-PCR using RT primer 21-5 and PCR primers 21-5 and 20-19 (or 20–27 for ACH) (see panel *A *for positioning). (***D***) RNAs from cellular clones isolated from four different infected patients and from MT4 cells were analyzed by a modified RT-PCR protocol using a PCR primer overlapping the SP1 splice junction. M = 100 bp marker (asterisk indicates the 600 bp band).

The sequencing of the 5'RACE products provided more information regarding the HBZ transcript. Indeed, the sequence data allowed us to demonstrate that alternative splicing of the RNA encoding HBZ was occurring. The antisense transcript initiating within the 3' LTR is spliced at two different positions (367 and 227 of the antisense strand) and joined to an internal region of the HBZ ORF at position 1767 (Fig. 4A). These HBZ RNA variants, which are referred to as spliced RNA 1 (SP1) and spliced RNA 2 (SP2), differ in the size of their exon 1 leading to an intronic region of 1400 nt and 1540 nt, respectively. Results of 5'RACE further suggested that the SP1 variant occurs more frequently than SP2.

Another important feature of the SP1 RNA was the presence of the splice acceptor downstream of the AUG initiation codon initially suggested by Gaudray *et al. *[11]. However, further analysis of the SP1 RNA sequence originating in the 3' LTR revealed a new in frame AUG initiation codon that permits proper initiation of HBZ translation (Fig. 4B). In contrast, no in frame AUG was identified within the HBZ SP2 RNA sequence flanking the splice junction and downstream of the first stop codon. It could however be possible that a non-AUG initiation codon (for example, GUG or CUG) could allow proper initiation of translation from this RNA. In fact, non-AUG initiation codons have been proposed for other HTLV-I proteins [23]. Amino acid sequence changes introduced limited variation in overall amino acid composition between these two potentially new HBZ isoforms and the previously published HBZ amino acid sequence [11]. For example, seven amino acids from the amino terminus of the original HBZ isoform would be substituted by four amino acids in the SP1-encoded isoform.

**Figure 5:**
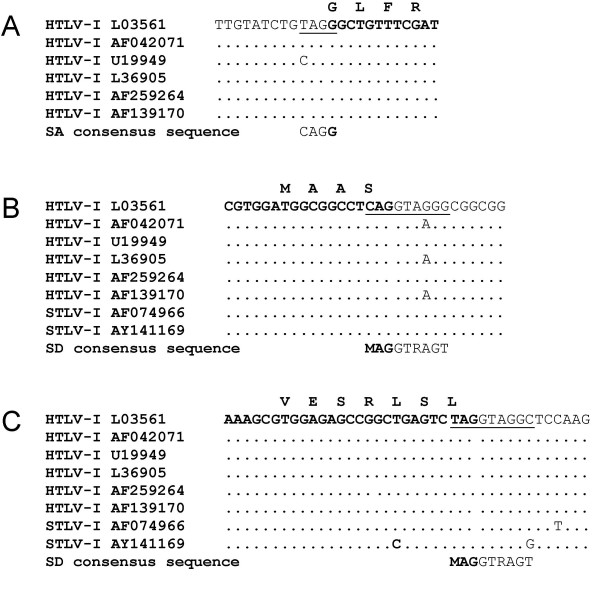
Sequence comparison of the HBZ splice acceptor, splice donors SD1 and SD2 and encoding regions between various HTLV-I and STLV-I isolates. STLV-I and HTLV-I sequences taken from GenBank were compared with different segments of the antisense strand of the K30 proviral DNA (accession number L03561): position 1756–1779 (splice acceptor) (**A**), position 350–379 (splice donor 1) (**B**) and position 182–239 (splice donor 2) (**C**). Comparisons were also made with the splice acceptor and splice donor consensus sequences (shown below compared stretches) and the corresponding K30 sequence is underlined. Coding regions are presented in bold and amino acid sequences are also indicated above the compared nucleotide sequence. GenBank accession numbers are provided for each compared STLV-I and HTLV-I proviral DNA clones.

Sequence analysis of the HTLV-I K30 proviral DNA revealed typical splice donor (SD) and splice acceptor (SA) consensus sequences at each end of the presumed intronic sequence for the predicted splice junction of both HBZ SP1 and SP2 RNAs (Fig. 5). Comparison with other HTLV-I sequences demonstrated strong conservation of the splice acceptor (Fig. 5A). Comparison of the SP1 SD sequence further indicated that this sequence was highly conserved in all HTLV-I and simian STLV-I LTR sequences analysed (Fig. 5B). In these sequence comparisons, it was noted that certain HTLV-I isolates in fact had a better match to the consensus sequence than the corresponding SD or SA sequence from the K30 proviral DNA clone. The SP2 SD sequence was also highly conserved among the various HTLV-I isolates, although certain isolates did present non-consensus SD sequences in this region (Fig. 5C and data not shown). In addition, comparison of LTR sequences from other HTLV-I and STLV-I isolates demonstrated a high degree of conservation within the predicted amino terminal sequences for both new HBZ isoforms (Fig. 5B–C).

To demonstrate that both HBZ splice variants existed in HTLV-I-infected and transfected cells, RT-PCR analysis was performed on isolated RNA with the forward primer 20-19 derived from the transcribed spliced 3' LTR and the reverse primer 21-5 located downstream of the identified splice acceptor (see Fig. 4A). This RT-PCR strategy was expected to generate a 684 bp signal for the HBZ SP1 RNA and a 544 bp signal for the HBZ SP2 RNA. Indeed for both tested HTLV-I-infected cell lines, i.e. C8166-45 and MJ, an amplified signal of the expected size for SP1 was present (Fig. 4C). However, the SP2 variant was only weakly detected in these infected cell lines. Similar analyses conducted in 293T cells transfected with K30, K30-3'/5681 and a different proviral DNA clone, i.e. ACH amplified the spliced HBZ SP1 and SP2 templates (very faint for SP2). Because of nucleotide sequence variation of the LTR region complementary to primer 20-19, the forward primer 20–27 (similar to the 20-19 primer, but with nucleotide sequence specificity for ACH) was used for RT-PCR analyses of ACH-transfected cells. To further demonstrate the existence of these spliced transcripts, the detection of HBZ spliced variants was evaluated in cell clones derived from HTLV-I-infected individuals (Fig. 4D). Taking in consideration the variability occurring in between HTLV-I isolates in the LTR region, primers from the HBZ-coding sequence that encompass the highly conserved splice junctions of SP1 and SP2 were used to detect antisense transcripts. Analysis of amplified products indeed demonstrated expression of the HBZ SP1 RNA variant in certain cell clones while other clones appeared negative. As a control, HTLV-I-infected MT4 cells were similarly analyzed and demonstrated amplification of the expected band. However, no signals were observed with primers overlapping the splice SP2 junction (data not shown).

These data thereby provide evidence for the existence of splicing events occurring in the HTLV-I antisense transcripts. A recent study has also confirmed the spliced nature of the HBZ RNA, having demonstrated the existence of the SP1 HBZ transcript [24]. In our study, we further suggest that, although the SP1 RNA variant represents the most abundant transcript, other spliced variants could exist (such as SP2). We have also importantly demonstrated that SP1 RNA variant is present in patient-derived cell clones, and unlike Satou *et al. *[24], not all tested cell clones were found to be positive for HBZ expression. Although more data is needed to understand the significance of these findings, these data might be indicative of a possible relationship between lack of HBZ expression and disease outcome. Furthermore, it is possible that the various identified HBZ RNA variants might contribute differently to HBZ protein synthesis. However, our PCR analysis has not permitted us to detect unspliced HBZ RNA in HTLV-I-infected cells or transfected 293T cells. Obviously, the designed PCR protocol used above favours shorther size PCR fragments derived from spliced HBZ RNA. Nonetheless, the formerly described HBZ isoform [11] could be produced from unspliced HBZ RNA although possible mechanisms might be needed for proper translation to occur from the resulting long 5' untranslated region of such a transcript. It should also not be excluded that other splice variants could also exist and contribute to post-transcriptional regulation of HBZ expression. Further experiments are presently underway to clearly establish if these other transcripts are indeed produced in infected cells.

### Positioning of the polyA addition site

**Figure 6:**
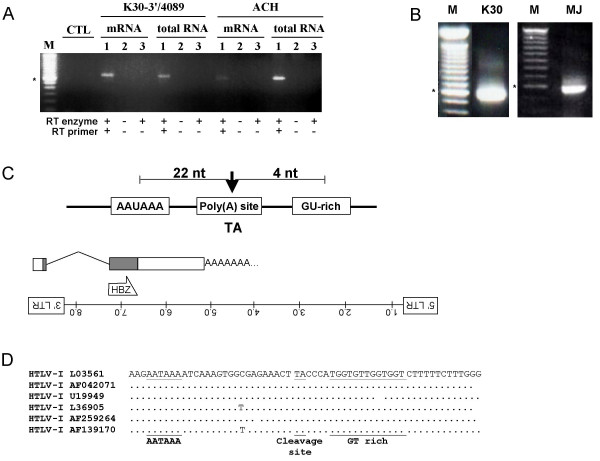
Identification of the polyA addition site of the HBZ transcript. (***A***), PolyA+ RNA and total RNA from 293T cells transfected with 5 μg K30-3'/4089 or ACH were analyzed by RT-PCR with the primers 21-5 and 20-19 (20–27 for ACH-transfected cells). Controls were performed for DNA contamination (lane 2) and autopriming (lane 3). CTL represents PCR amplification conducted in the absence of cDNA or RNA samples. M = 100 bp marker (the asterisk indicates the 600 bp band). (***B***) RNA samples from 293T cells transfected with 5 μg K30 or HTLV-I-infected MJ cells were analysed by 3' RACE. Amplified products were run next to a 100 bp marker (M). (***C***) Position of the polyA addition site (indicated with arrow) next to a consensus polyA signal and a GU-rich consensus sequence. The structure of the HBZ mRNA with the most representative HBZ spliced variant (SP1) and the 3' polyA tail is shown below. Dark boxes represent the coding portion of the transcript. The complete proviral DNA and the former HBZ ORF are also shown below. **(*D*) **HTLV-I sequences taken from GenBank were compared with polyA signals (position 3821–3880) located on the antisense strand of the K30 proviral DNA (accession number L03561). Comparisons were focussed on the AATAAA polyA signal, the cleavage site deduced from our 3'RACE results and the GT-rich sequence (underlined in the K30 proviral DNA sequence). GenBank accession numbers are provided for each compared HTLV-I proviral DNA clones.

We next sought to demonstrate that the HBZ transcript was polyadenylated. A potential polyA signal has previously been suggested to direct the addition of a polyA tail to the 3' end of the HTLV-I antisense transcript [4]. Therefore, a variant of the K30-3'/5681 construct that includes this potential polyA signal was generated (K30-3'/4089). This new construct and the ACH proviral DNA were transfected into 293T cells. An SP1-derived signal was observed in both transfected cells following analysis of total RNA or mRNA using the RT-PCR approach described above (Fig. 6A), thereby demonstrating that this transcript was polyadenylated. The SP2-specific band was generally too weak to be easily detected in these analyses. The polyA addition site was precisely mapped using 3'RLM-RACE to specifically amplify the 3' end of polyadenylated RNA. RNA extracted from 293T cells transfected with K30 or from HTLV-I-infected MJ cells was used for the 3'RACE analysis. Initial analysis using a primer positioned downstream of the HBZ stop codon amplified a 600 bp fragment from both RNA samples (Fig. 6B). Sequencing of this fragment demonstrated that the polyA tail was positioned 1450 nt from the HBZ stop codon. The polyA addition site was located in a UA dinucleotide positioned 22 nucleotides downstream of the previously suggested polyA signal and a few nucleotides from a GU-rich segment, another typical consensus sequence for polyA addition [25] (Fig. 6C). These consensus sequences were highly conserved among other HTLV-I proviral DNAs (Fig. 6D).

These results hence have permitted to identify the 3'end of the spliced HBZ transcript. Taking into account the results of Fig. 4, we predict the size of the more abundant HBZ SP1 transcript to be 2.4 kb. This characterization of the HTLV-I antisense transcript hence agrees with previous findings of Larocca *et al.*, who detected a 2.5 kb antisense transcript [4]. Our results also confirm the Northern blot data of this former study as to the possible existence of an intron at a similar position in the antisense transcript of HTLV-I. Furthermore, presence of the 3' untranslated region might suggest a potential role for this region in post-transcriptional regulation of HBZ expression. Further experiments will be needed to assess this possibility.

### Synthesis of the various HBZ isoforms

**Figure 7:**
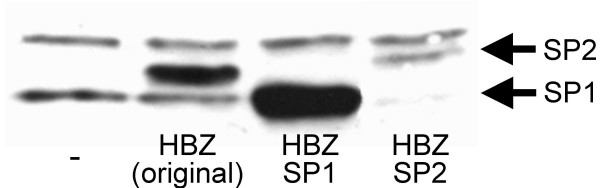
Synthesis of the various HBZ isoforms. Cell extracts were prepared from 293T cells transfected with 4 μg pcDNA3.1-Myc-His HBZ, pcDNA3.1-Myc-His HBZ SP1, pcDNA3.1-Myc-His HBZ SP2 or the empty vector (-). HBZ isoforms were detected by Western blot using anti-Myc antibodies. The position of the SP1- and SP2-derived HBZ isoforms is indicated by arrows.

Based on our data demonstrating the existence of differently spliced HBZ RNA, different HBZ isoforms could be expressed in HTLV-I-infected cells. However, the HBZ SP2 RNA appeared as a weak signal and depended on a non-AUG initiation codon. To confirm the translation of both isoforms, complete cDNAs (including the 5' untranslated region determined from our 5'RLM-RACE data) were amplified for each splice variant and tagged with the Myc epitope by cloning into the pcDNA3.1-Myc-His A expression vector. These constructs, and a vector expressing the originally published HBZ isoform [20], were transfected into 293T cells and detected by Western blot with a mouse anti-Myc antibody. Both new HBZ isoforms were detected in transfected 293T cells and the HBZ isoform produced from the SP1 cDNA had a lower molecular weight than either the original or the SP2 HBZ isoforms (Fig. 7). Although the position of the initiation codon was not determined for the HBZ SP2 isoform, the estimated size of the protein suggested that translation initiation occurred within exon 1. Immunofluorescent analysis of the transfected cells demonstrated nuclear localization of the two new HBZ isoforms, as described for the original HBZ protein (data not shown) [26].

**Figure 8:**
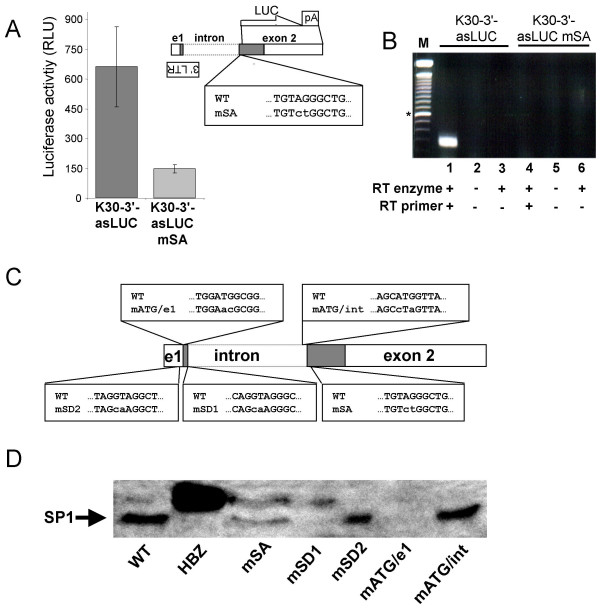
Importance of the SD/SA sequences and of the SP1-specific ATG for HBZ protein synthesis (***A***) 293T cells were co-transfected with 5 μg K30-3'-asLUC or K30-3'-asLUC mSA and 2 μg pActin-β-gal. Luciferase activities represent the mean value of three measured samples ± S.D and are expressed as normalised RLU for 5 × 10^6 ^cells. **(*B*)**. 293T cells were co-transfected with 5 μg K30-3'-asLUC or K30-3'-asLUC mSA and 2 μg pActin-βgal. RNA samples from transfected cells were analysed by a modified RT-PCR protocol (see Materials and Methods). Controls for DNA contamination (lanes 2 and 5) and autopriming (lanes 3 and 6) were included. M = 100 bp marker (the asterisk indicates the 600 bp band). **(*C*) **The K30-3'/4089 construct was mutated at the splice acceptor (mSA), the splice donor of SP1 (mSD1), the splice donor of SP2 (mSD2), the presumed ATG initiation codon of SP1 (mATG/e1) or the initially identified ATG initiation codon (mATG/int). Comparison of sequences between wild-type and mutated versions of K30-3'/4089 are depicted. (***D***) 293T cells were transfected with 2 μg pActin-β-gal and 5 μg pcDNA3.1-Myc-His HBZ, wild-type K30-3'/4089 or versions mutated for SA, SD1, SD2, ATG/e1 or ATG/int and nuclear extract from samples transfected with equal efficiency (based on β-gal read-outs) were analysed by Western blot using anti-HBZ antiserum. The position of the SP1-specific HBZ isoform is indicated by an arrow.

The importance of splicing events for HBZ protein synthesis was next determined by generating a K30-3'/5681 construct (termed K30-3'-asLUC) in which the sequence downstream of the splice acceptor was replaced with an SV40 polyA signal and the luciferase reporter gene positioned in frame with the rest of the HBZ amino acid sequence. This construct provided a reliable and sensitive tool for quantification of HBZ transcription. Using the wild-type or a SA-mutated version of K30-3'-asLUC, the importance of the SA consensus sequence was then assessed by co-transfection experiments. Results presented in Fig. 8A indicated that mutation of the splice acceptor significantly reduced luciferase activity below that of the wild type vector in transfected 293T cells. RT-PCR analyses using primers derived from the luciferase gene and the 3' LTR confirmed the production of a spliced RNA from the wild type construct while no specific signals were observed in RNA samples from cells transfected with the mutated K30-3'-asLUC vector (Fig. 8B).

To confirm these data and extend our analyses to other splice consensus sequences and to the two different possible AUG initiation codon, mutations of the K30-3'/4089 construct specifically targeting SD/SA consensus sequences, as well as both putative AUG translation initiation codons, were specifically generated (Fig. 8C). Following transfection of wild-type and mutated K30-3'/4089 constructs into 293T cells, the HBZ protein was detected by Western blot (Fig. 8D). Significantly less HBZ protein was detected when the proviral DNA was mutated in the SA or SP1 SD sequence, or the SP1-specific AUG, suggesting that SP1 mRNA is important for HBZ protein synthesis. On the other hand, mutation of the intronic AUG or the SP2 SD sequence had little impact on HBZ protein levels. Interestingly, transfection of 293T cells with a vector expressing the original HBZ isoform produced HBZ protein of a higher molecular weight than K30 HBZ protein, which may depend on presence of the Myc tag and differences in amino terminus.

These data indeed suggested the possible existence of different HBZ isoforms. In agreement with our RT-PCR analysis, our results suggest that the SP1 RNA-translated HBZ isoform contributes importantly to overall HBZ protein synthesis. It should be noted that, in our Western blot analyses, a constant shift in migration of the SP1-derived isoforms is observed when compared to the other HBZ isoforms. Although these results are unexpected given the small differences in amino acid composition between the various HBZ isoforms, we could speculate that the SP1 isoform is differently modified at a post-translational level, which would then account for these suggested variations. Further experiments are needed to address this issue.

### Functional properties of the SP1 RNA-derived HBZ isoform

**Figure 9:**
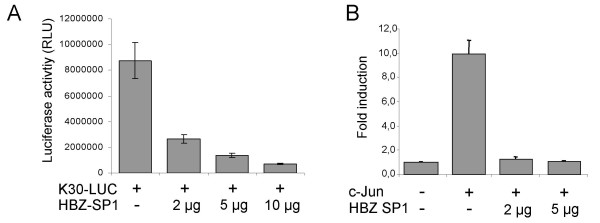
Functional properties of the SP1-derived HBZ isoform. (***A***) 293T cells were co-transfected with 2 μg of K30-LUC and increasing concentrations of pcDNA3.1-Myc-His HBZ SP1 Δ 5'UTR, along with the β-gal reporter vector. **(*B*) **CEM cells were co-transfected with the collagenase promoter-driven luciferase reporter construct (2 μg), pcDNA-c-Jun (1 μg), pcDNA3.1-Myc-His HBZ SP1 Δ 5'UTR (2 and 5 μg), and the β-gal reporter vector (5 μg). Luciferase activities represent the mean value of three measured samples ± S.D and are expressed as normalised RLU for 5 × 10^6 ^cells. Fold inductions in panel *B *were calculated with respect to CEM cells transfected in the absence of pcDNA-c-Jun (set at a value of 1).

Since these data suggested that the HBZ SP1 mRNA was the most abundant HBZ transcript and contributed significantly to HBZ protein synthesis, we next determined whether the SP1-encoded HBZ protein had similar effects on transcription as described for the original HBZ protein [11,18,19]. The effect of the HBZ SP1 isoform on HTLV-I LTR activity was tested in the context of a complete proviral DNA containing a luciferase reporter gene inserted in frame with the envelope amino acid sequence. Transfection of the SP1 expression vector into 293T cells significantly reduced luciferase activity (Fig. 9A). The effect of the HBZ SP1 isoform on c-Jun-dependent transcriptional activation was also evaluated by co-transfecting CEM cells with HBZ SP1 and c-Jun expression vectors along with a collagenase promoter driving luciferase gene expression. The HBZ SP1 expression vector strongly reduced c-Jun-mediated induction of luciferase activity (Fig. 9B), arguing strongly that the SP1-derived HBZ isoform possesses a transcriptional inhibitory function similar to the original HBZ isoform. These data again reinforce the notion that the major HBZ isoform should act similarly as to the originally presented HBZ isoform and might thus play an important role in HTLV-I latency.

In this study, we have thoroughly characterized the antisense transcripts produced from the HTLV-I retrovirus and responsible for the synthesis of the previously described HBZ protein. Using different RT-PCR approaches, our results first demonstrated that antisense transcripts could be detected in HTLV-I-infected cell lines and 293T cells transfected with proviral DNA and initiated in the R and U5 segments of the LTR. Transcripts were alternatively spliced at a varying frequency and produced two new isoforms with translation initiating in exon 1, at least for the most abundant variant. PolyA site was positioned at a distance of 1450 nt form the HBZ stop codon and occurred next to known polyA signals. Mutation experiments also showed the importance of the SP1 mRNA for HBZ protein synthesis. Transfection experiments also indicated that the isoform produced from HBZ SP1 mRNA demonstrated suppression of AP-1- and Tax-dependent transcriptional activation.

Our results strongly argue that the major spliced antisense transcript is responsible for producing the HBZ protein. However, the minor spliced form and the unspliced HBZ transcript may be important sources of HBZ expression in other cellular contexts or states. More data are needed to indeed confirm that the SP2 transcript is indeed produced in several other HTLV-I-infected cells and that both SP2- and unspliced derived HBZ isoforms can be detected at the protein level in infected cells. In light of the possible existence of multiple HBZ RNA variants, it could then be postulated that transcriptional and post-transcriptional mechanisms might regulate HBZ mRNA and protein levels and drive the type of transcript (and isoform) being produced. These mechanisms might involve other HTLV-I viral proteins. Regulation of HBZ protein levels and functions will likely modulate HTLV-I latency and pathogenesis. Detection of varying levels of the major spliced form of HBZ RNA in several cellular clones isolated from infected patients (even in the same patient) is highly relevant in this regard. Future investigations will need to address the different mechanisms regulating HBZ protein synthesis.

## Conclusion

Our study has an important impact on the field of retrovirology, in general. These data provide the strongest evidence for the existence of retroviral antisense transcripts, which have previously been seen as potential artefacts. It is likely that antisense transcripts are also produced in other retroviruses (human and non-human) and could encode for proteins as previously proposed for HIV-1 and FIV [5,8,22,27]. Based on our data, further studies on antisense transcription are warranted, specifically in complex retroviruses. The presence of one or more potentially new genes in these transcripts would provide important new insights into retroviral regulation and function, resulting in a more complete understanding of these viruses. It will be of great interest to determine whether regulatory processes linked to antisense transcription are active in HTLV-I, such as the antisense effect previously suggested for these transcripts in HIV-1 [28,29].

## Methods

### Cell lines and antibodies

All T-cell lines were maintained in RPMI-1640 culture medium supplemented with 10% fetal bovine serum (Hyclone Laboratories, Logan, UT), 2 mM glutamine, 100 U/ml penicillin G, and 100 μg/ml streptomycin. 293T cells were grown in supplemented DMEM. Peripheral blood mononuclear cells (PBMCs) from HTLV-I-infected individuals were cloned by limiting dilution (0.1 cell per well) in the presence of feeder cells (γ-irradiated allogeneic PBMCs (5 × 10^5 ^cells/ml)) and in complete RPMI 1640 containing 10% filtered human serum AB, recombinant IL-2 (100 U/ml), PHA (1 μg/ml). Positive cultures were transferred into 96 U-bottom plates and stimulated every 14 days with PHA and fresh feeder cells (1 × 10^6 ^cells/ml). Derived cellular clones were identified as YB034 to YB356 (patient 1), J1+ (patient 2), 1P8 (patient 3) and Jas081 (patient 4). The anti-HBZ antiserum has been described previously [11]. Mouse anti-Myc antibody 9E10 was purchased from Sigma. Goat anti-mouse and anti-rabbit IgG antibodies coupled to the horse radish peroxidase were obtained from Amersham Bioscience.

### Vectors and site-directed mutagenesis

HTLV-I proviral DNA constructs used in this study were ACH [30] and K30 [31]. The K30-3'/5681 and K30-3'/4089 constructs were derived from K30 DNA by subcloning 3' segments (positions 5681 to 9043 and 4089 to 9043, respectively) in pBlueScript KS. The K30-LUC proviral DNA construct contains the luciferase reporter gene cloned in frame to the ATG initiation codon of the envelope gene has been previously reported [32]. The K30-3'-asLUC construct was generated from the K30-3'/5681 vector by introducing a NcoI site at position 1791 (antisense strand) located in the HBZ-coding region and downstream of the splice acceptor with the QuikChange XL Site-Directed Mutagenesis Kit (Stratagene) and the primer 5'-GCTTGCCTGTGA**CCATGG**CCGGAGGACCTGC-3' and the complementary primer. A luciferase reporter gene/SV40 polyA cassette isolated from pGL3-Basic (Promega) was cloned in the NcoI/SalI sites concomitantly deleting the sequence positioned downstream of the mutated region of the HBZ ORF. Mutagenesis of the splice acceptor region at position 1766 (antisense strand) was similarly conducted using the primer 5'-CTTTGTATCTGT**CT**GGCTGTTTCGATGCTTGCCTG-3' (with the mutated sequence indicated in bold) generating K30-3'-asLUC mSA. Other mutagenesis strategies were undertaken in the K30-3'/4089 construct in order to mutate the splice acceptor (as indicated above), splice donor 1 (position 368; 5'-GGCGGCCTCAG**CA**AGGGCGGCGGG-3'), splice donor 2 (position 228; 5'-GCCGGCTGAGTCTAG**CA**AGGCTCCAAGGG-3'), the intronic ATG (position 1746; 5'-GTGGGCTGATAATAAGC**C**T**A**GTTAACTTTGTATCTG-3') and the exon 1 ATG (position 356; 5'-CAACCGGCGTGGA**AC**GCGGCCTCAGGTAGGG-3'). The pActin-β-gal vector contains the β-galactosidase gene under the control of the β-actin promoter. SP1 and SP2 HBZ cDNAs (including the 5' untranslated region (UTR)) were amplified and cloned in the pcDNA3.1-Myc-His A expression vector generating pcDNA3.1-Myc-His HBZ SP1 and pcDNA3.1-Myc-His HBZ SP2, respectively. An equivalent construct bearing the HBZ SP1 cDNA without the 5' UTR was also produced (pcDNA3.1-Myc-His HBZ SP1 Δ 5'UTR). The construct expressing a Myc-tagged version of the former HBZ isoform (pcDNA3.1-Myc-His HBZ), the collagenase promoter-luciferase and pcDNA3.1-c-Jun vectors have been previously described [18,20].

### Transfection and gene reporter assays

293T cells were transfected with 5–10 μg of DNA through the calcium phosphate protocol as previously described [33]. CEM cells were transfected according to a previously described protocol [34]. In transfection experiments with K30-LUC or collagenase promoter-luciferase vectors, the pcDNA3.1-Myc-His A empty vector was used to standardize DNA quantity in between transfection samples. Transfected cells were lysed 48 hours post-transfection in a lysis buffer (25 mM Tris phosphate, pH 7.8, 2 mM DTT, 1% Triton X-100, 10% glycerol) and luciferase activity read out was performed with the MLX microplate luminometer (Dynex Technologies) with a single injection of a luciferase buffer (20 mM tricine, 1.07 mM (MgCO_3_)_4_·Mg(OH)_2_·5H_2_O, 2.67 mM MgSO_4_, 0.1 mM EDTA, 220 μM Coenzyme A, 4.7 μM D-Luciferin potassium salt, 530 μM ATP, 33.3 mM DTT). Each sample was co-transfected with a β-gal-expressing vector for normalisation. The β-galactosidase activity was measured using the Galacto-Light™ kit (Applied Biosystems, Bedford, MS) according to manufacturer's suggestions. Luciferase activity are presented in Relative Light Units (RLU) and represent the calculated mean ± SD of three transfected samples normalised by the measured β-galactosidase activity.

### RT-PCR and 5'/3' RACE analyses

Total RNA was extracted by the Trizol reagent (Invitrogen) from HTLV-I-infected cell lines or transfected 293T cells. PolyA+ RNA was purified from lysed cell samples using the Poly(A)Purist™ Kit (Ambion) and according to manufacturer's instructions. RT-PCR analyses were conducted using RT primer 21-5 (5'-AACTGTCTAGTATAGCCATCA-3'). Prior to RT, RNAs were treated with DNAseI and incubated at 70°C for 5 min. RNA (5 ug) was then added to 1.5 μM RT primer, 1 mM dNTPs, 15 U AMV reverse transcriptase (USB), 1× AMV Reaction buffer and 10 U SUPERase·In RNAse inhibitor (Ambion) and RT reactions were incubated for 2 hours at 42°C. Aliquots from the RT reactions were then PCR amplified in the presence of 1.25 U Taq DNA polymerase (New England BioLabs Inc.), 1× ThermoPol buffer, 20 μM dNTP, 1.5 uM of each primer and 4% DMSO using a Tgradient thermocycler (Biometra). Primers added to the PCR reactions were the reverse 21-5 primer and the forward primer 21-4 (5'-TGCTGGTGGAGGAATTGGTGG-3'), 23-3 (5'-CAAGGAGGAGGAGGAAGCTGTGC-3'), 20-19 (in the 3' LTR: 5'-CGCAGAGTTGAACAAGCAGG-3') and 20–27 (in the 3' LTR specific for ACH: 5'-CGCAGAGGTGAGCAAACAGG-3'). PCR conditions were as follow: a first step of denaturation at 94°C for 5 min followed by 35 to 40 cycles of denaturation (94°C for 1 min.), annealing (60°C for 1 min.) and extension (72°C for 1 min.) and a final extension at 72°C for 5 min. In RT-PCR analyses of the cellular clones isolated from infected patients, a modified RT-PCR approach designed to reduce autopriming was carried out using the RT primer 42-5 consisting of a HBZ-specific 3' end (in bold) and a non-hybridizing 5' end (5'-AGTAGAGTATCGACGATACA**CAACTGTCTAGTATAGCCATCA**-3) followed by PCR amplification with reverse primer 24-21 (specific to the 5' end of the RT primer: 5'-AGTAGAGTATCGACGATACACAAC-3') and forward primer 18-4 (5'-ATGGCGGCCTCAGGGCT-3') (overlapping the SP1 splice junction). For amplification of the SP2 spliced variant, in these experiments, primer 18-4 was substituted by forward primer 19-19 (5'-CGGCTGAGTCTAGGGCTGTTT-3') during PCR amplification. A similar strategy was used for RT-PCR analysis of transfection experiment with the different K30-3'-asLUC constructs, in which the RT primer was 49-1 (luciferase-specific 3' end in bold) (5'-CCATCATCACATTGGAATATC**GCCTTTCTTTATGTTTTTGGCGTCTTCC**-3) and forward and reverse primers for PCR were 20-19 and 25–30 (specific to the 5'end of the RT primer: 5'-AGTAGAGTATCGACGATACACAAC-3') respectively. RT-PCR amplifications were controlled for DNA contamination (RNA samples with no RT step) and autopriming (cDNA synthesis reaction in the presence of RT with no added specific primer). Extremities of the HBZ RNA (5' and 3') were analyzed from isolated total RNA with the FirstChoice RLM-RACE kit from Ambion according to the manufacturer's instructions. For the 5'RACE protocol, cDNAs were synthesized with random decamers and the subsequent two PCR rounds were conducted with the supplied 5'RACE outer and inner primers and HBZ-specific primers 21-9 (5'-TCCTCTTTCTCCCGCTCTTTT-3') and 20-18 (5'-CCGCGGCTTTCCTCTTCTAA-3') successively. For the 3'RACE protocol, cDNA synthesis was performed in the presence of the supplied 3'RACE adapter; PCR amplification was achieved through 3'RACE inner and outer primers and primers 24-20 (5'-CGAGGATGTGGTCTAGGTTAGA-3') and 22-15 (5'-GGCTGGGTTCGGTATTAAGGAA-3') derived from the sequence downstream of the HBZ stop codon. Amplified products were then directly sequenced or first cloned in pBlue Script KS+ (Stratagene) before sequencing.

### Western blot analysis

Transfected 293T cells were lysed and total protein or nuclear extracts were prepared as previously described [26,35]. Equal quantities of extracts were run on a SDS-12% PAGE and transferred to PVDF membranes (Millipore). The blot was next blocked in PBS 1X/5% milk and incubated with a mouse anti-Myc 9E10 antibody (dilution 1/1000) or anti-HBZ antiserum (dilution 1/1000). After several washes, signals were revealed by the addition of peroxydase-conjugated goat anti-mouse IgG (dilution 1/2000) or goat anti-rabbit IgG (dilution 1/10000) antibodies and subsequent incubation with the ECL reagent (Amersham Pharmacia Biotech). Membranes were exposed on hyperfilms ECL (Amersham Pharmacia Biotech).

## List of abbreviations

HBZ: HTLV-I bZIP

HIV-1: human immunodeficiency virus type 1

HTLV-I: human T-cell leukemia virus type I

LTR: long terminal repeat

## Competing interests

The author(s) declare that they have no competing interests.

## Authors' contributions

MHC carried out most of the RT-PCR analyses, the 5'and 3' RACE analyses, mutagenesis of the proviral DNA clones and drafted the manuscript. SL has performed and designed a number of RT-PCR experiments, has helped in conducting sequence alignment and Western blot analysis of the transfected mutants. BA, CAA and PH have performed transfection experiments, luciferase assay and Western blot analysis. MEP has helped in sequence alignment and has prepared several proviral DNA constructs. JT has conducted the RT-PCR analyses from the patient's cell clone. EW has participated in the design of these analyses and has helped in drafting the manuscript. SJM has helped in drafting and finalizing the manuscript and has provided important input on the design of the study. JMM and BB have conceived the study, participated in its coordination, helped in drafting the manuscript and finalizing the manuscript. All authors read and approved the final manuscript.

## References

[B1] Lavorgna G, Dahary D, Lehner B, Sorek R, Sanderson CM, Casari G (2004). In search of antisense. Trends Biochem Sci.

[B2] Vanhee-Brossollet C, Vaquero C (1998). Do natural antisense transcripts make sense in eukaryotes?. Gene.

[B3] Knee R, Murphy PR (1997). Regulation of gene expression by natural antisense RNA transcripts. Neurochem Int.

[B4] Larocca D, Chao LA, Seto MH, Brunck TK (1989). Human T-cell leukemia virus minus strand transcription in infected T-cells. Biochem Biophys Res Commun.

[B5] Vanhee-Brossollet C, Thoreau H, Serpente N, D'Auriol L, Levy JP, Vaquero C (1995). A natural antisense RNA derived from the HIV-1 env gene encodes a protein which is recognized by circulating antibodies of HIV+ individuals. Virology.

[B6] Peeters A, Lambert PF, Deacon NJ (1996). A fourth Sp1 site in the human immunodeficiency virus type 1 long terminal repeat is essential for negative-sense transcription. J Virol.

[B7] Michael NL, Vahey MT, d'Arcy L, Ehrenberg PK, Mosca JD, Rappaport J, Redfield RR (1994). Negative-strand RNA transcripts are produced in human immunodeficiency virus type 1-infected cells and patients by a novel promoter downregulated by Tat. J Virol.

[B8] Miller RH (1988). Human immunodeficiency virus may encode a novel protein on the genomic DNA plus strand.. Science.

[B9] Bukrinsky MI, Etkin AF (1990). Plus strand of the HIV provirus DNA is expressed at early stages of infection. AIDS Res Hum Retroviruses.

[B10] Bentley K, Deacon N, Sonza S, Zeichner S, Churchill M (2004). Mutational analysis of the HIV-1 LTR as a promoter of negative sense transcription. Arch Virol.

[B11] Gaudray G, Gachon F, Basbous J, Biard-Piechaczyk M, Devaux C, Mesnard JM (2002). The complementary strand of the human T-cell leukemia virus type 1 RNA genome encodes a bZIP transcription factor that down-regulates viral transcription. J Virol.

[B12] Miyoshi I, Kubonishi I, Yoshimoto S, Akagi T, Ohtsuki Y, Shiraishi Y, Nagata K, Hinuma Y (1981). Type C virus particles in a cord T cell line derived by co-cultivating normal human cord leukocytes and human leukaemic T cells.. Nature.

[B13] Poiesz B, Ruscetti FW, Gazdar AF, Bunn PA, Minna JD, Gallo RC (1980). Detection and isolation of type C retrovirus particles from fresh and cultured lymphocytes of a patient with cutaneous T-cell lymphoma.. Proc Natl Acad Sci USA.

[B14] Yoshida M, Miyoshi I, Hinuma Y (1982). Isolation and characterization of retrovirus from cell lines of human adult T-cell leukemia and its implication in the disease.. Proc Natl Acad Sci USA.

[B15] Gessain A, Barin F, Vernant JC, Gout O, Maurs L, Calender A, de The G (1985). Antibodies to human T-lymphotropic virus type-I in patients with tropical spastic paraparesis. Lancet.

[B16] Osame M, Usuku K, Izumo S, Ijichi N, Amitani H, Igata A, Matsumoto M, Tara M (1986). HTLV-I associated myelopathy, a new clinical entity. Lancet.

[B17] Rodgers-Johnson P, Gajdusek DC, Morgan OS, Zaninovic V, Sarin PS, Graham DS (1985). HTLV-I and HTLV-III antibodies and tropical spastic paraparesis. Lancet.

[B18] Basbous J, Arpin C, Gaudray G, Piechaczyk M, Devaux C, Mesnard JM (2003). The HBZ factor of human T-cell leukemia virus type I dimerizes with transcription factors JunB and c-Jun and modulates their transcriptional activity. J Biol Chem.

[B19] Matsumoto J, Ohshima T, Isono O, Shimotohno K (2005). HTLV-1 HBZ suppresses AP-1 activity by impairing both the DNA-binding ability and the stability of c-Jun protein. Oncogene.

[B20] Thebault S, Basbous J, Hivin P, Devaux C, Mesnard JM (2004). HBZ interacts with JunD and stimulates its transcriptional activity. FEBS Lett.

[B21] Arnold J, Yamamoto B, Li M, Phipps AJ, Younis I, Lairmore MD, Green PL (2006). Enhancement of infectivity and persistence in vivo by HBZ, a natural antisense coded protein of HTLV-1. Blood.

[B22] Briquet S, Richardson J, Vanhee-Brossollet C, Vaquero C (2001). Natural antisense transcripts are detected in different cell lines and tissues of cats infected with feline immunodeficiency virus. Gene.

[B23] Corcelette S, Masse T, Madjar JJ (2000). Initiation of translation by non-AUG codons in human T-cell lymphotropic virus type I mRNA encoding both Rex and Tax regulatory proteins. Nucleic Acids Res.

[B24] Satou Y, Yasunaga J, Yoshida M, Matsuoka M (2006). HTLV-I basic leucine zipper factor gene mRNA supports proliferation of adult T cell leukemia cells. Proc Natl Acad Sci U S A.

[B25] Zhao J, Hyman L, Moore C (1999). Formation of mRNA 3' ends in eukaryotes: mechanism, regulation, and interrelationships with other steps in mRNA synthesis. Microbiol Mol Biol Rev.

[B26] Hivin P, Frederic M, Arpin-Andre C, Basbous J, Gay B, Thebault S, Mesnard JM (2005). Nuclear localization of HTLV-I bZIP factor (HBZ) is mediated by three distinct motifs. J Cell Sci.

[B27] Briquet S, Vaquero C (2002). Immunolocalization studies of an antisense protein in HIV-1-infected cells and viral particles. Virology.

[B28] Tagieva NE, Vaquero C (1997). Expression of naturally occurring antisense RNA inhibits human immunodeficiency virus type 1 heterologous strain replication. J Gen Virol.

[B29] Kim JH, McLinden RJ, Mosca JD, Vahey MT, Greene WC, Redfield RR (1996). Inhibition of HIV replication by sense and antisense rev response elements in HIV-based retroviral vectors. J Acquir Immune Defic Syndr Hum Retrovirol.

[B30] Kimata JT, Wong FH, Wang JJ, Ratner L (1994). Construction and characterization of infectious human T-cell leukemia virus type 1 molecular clones. Virology.

[B31] Zhao TM, Robinson MA, Bowers FS, Kindt TJ (1995). Characterization of an infectious molecular clone of Human T-cell Leukemia Virus type I.. J Virol.

[B32] Langlois M, Audet B, Legault E, Pare ME, Ouellet M, Roy J, Dumais N, Mesnard JM, Rothstein DM, Marriott SJ, Tremblay MJ, Barbeau B (2004). Activation of HTLV-I gene transcription by protein tyrosine phosphatase inhibitors. Virology.

[B33] Fortin JF, Cantin R, Lamontagne G, Tremblay M (1997). Host-derived ICAM-1 glycoproteins incorporated on human immunodeficiency virus type 1 are biologically active and enhances viral infectivity. J Virol.

[B34] Lemasson I, Thebault S, Sardet C, Devaux C, Mesnard JM (1998). Activation of E2F-mediated transcription by human T-cell leukemia virus type I Tax protein in a p16(INK4A)-negative T-cell line. J Biol Chem.

[B35] Schreiber E, Matthias P, Müller M, Schaffner W (1989). Rapid detection of octamer binding proteins with 'mini-extract' prepared from a small number of cells.. Nucleic Acids Res.

